# Supplementary material for: Exploring proxies for occupation intensity in hunter-gatherer settlement systems: A combination of ethnohistoric and archaeological data
Source: PLoS One. 2025 Nov 7;20(11):e0333870. doi: 10.1371/journal.pone.0333870 (PMC12594338; doi:10.1371/journal.pone.0333870)
Supplement: S1 File — (DOCX) [file pone.0333870.s001.docx]

**Supporting Information:**

***Exploring proxies for occupation intensity in hunter-gatherer settlement systems: A combination of ethnohistoric and archaeological data***

**S1: Supporting Information**

Ground stone assemblage details

Ground stone implements in the Sierra Pinacate are nearly exclusively bottom stones: mortars, metates, and amorphous forms too large for use as hand stones. The most unique form is the gyratory crusher, which is a mortar with a hole in the bottom of the implement inferred to be used with a specialized pestle. We believe most of these first completed a use-life as standard mortars until a hole was worn in the bottom. The lack of top stones (manos, pestles, etc.) suggests the use of wooden implements. The Las Playas assemblages included a range of tools associated with shell jewelry manufacture for export, i.e. abraders that are absent in the Sierra Pinacate. This captures an additional aspect of the economy and emphasizes the point that hunters-gatherers rarely lived in a societal vacuum. Subsistence tasks at coastal locations were dominated by activities distinct from the Sierra Pinacate and Las Playas sites but ground stone was present at most shell mounds. The large La Morúa site contained 53 implements in 13% of the site’s total area, suggesting an assemblage size of 407 could be present. We use this estimated number in the data presented in Fig 5.

Ground stone was typically exhaustively recorded at all sites. The site of Cuervos may reflect an undercounting of implements as a portion was mapped only with a drone. Estimating based on the density of ground stone at similar sites, there may be as many 27 specimens lacking from the site count. Making this correction would have the effect of strengthening the correlation between ground stone counts and structure counts and further illustrating the lack of correlation between site area and ground stone counts. The data presented in the principal text are not corrected in this manner.

Given that 78.6 percent of the Sierra Pinacate ground stone assemblage was broken, we suspect a cultural ethic to destroy implements at the death of the user. Alternatively, inter-group hostility potentially targeted useful site furniture. Whole and broken ground stone objects were frequently incorporated into windbreak foundations. It is possible that some whole implements were removed over the last ~100 years by neighboring populations for reuse. In general, though, the domestic nature of ground stone and its weight—and difficulty to transport—make it a better proxy of occupation intensity than hunting implements such as projectile points, which are more likely to be lost outside of habitation areas [cf. 1].

In the primary text we employ a simple count of implements. We explored alternative approaches intended to be more precise to account for the tendancy of groundstone to fracture overtime and thus inflate the count. Additionally, as we ultimately seek to track person hours spent on site, the amount of use evidenced by each piece is potentially a more sensitive metric. In the end, the correlation between alternative methods was so strong that the more complicated calculations seem to add complexity without any additional value and thus are not discussed in the principal text.

Ground stone quantification

The following approaches to quantification were considered:

“All ground stone” is a simple count of major implements (complete and incomplete).

“All bottoms” refers to a count of all implements used as stationary grinding surfaces (netherstones). This is the method utilized in the primary text.

“Sum bottom” totals site/loci assemblages by the total proportion of bottom stones present as visually estimated in intervals of 10%. For example, a metate estimated at 50% would be counted as .5 in the summation of site groundstone.

“Sum wear mm” totals estimated wear depths based on a 4 level scale particular to three morphologies (flat, basin, mortar-bowl) times the proportion of the implement present. For example, the four levels of mortar wear correspond to 10, 30, 50, and 125 mm of depth. A few exceptional pieces were assigned individual values. The resulting metric is thus in increments of millimeters.

In S1 Table we provide the correlation coefficient for these alternative methods highlighting the overall utility of the simple count of implement methods.

Alternative approaches to dealing with 0 data in the ground stone counts

As explained in text, our distributional fit analyses followed prior applications, most notably Haas et al. [2]. However, we also experimented with other derivations which we found informative of the structure of our data and sampling biases likely common to most settlement survey data. Specifically, our data exploration highlights one explanation for why power-laws may be difficult to observe in artifact data across the full range of values, as opposed to only upper tails. In addition to adding a value of one to the entire range of values in a distribution, we also ran iterations that raised the count only of sites with zero ground stone to one. This had the effect of approximately doubling the counts of one-ground stone implement sites (0 and 1 values were approximately equally abundant). This approach produced a high p value in the Power-Law test where the standard methodology of adding one to every value of the data set (not just to zero values) did not support a power-law distribution as plausible. As noted in primary text testing, only the upper tail did produce a statistically significant result.

On one hand, our results offer confirmatory evidence that the upper tails of the distribution are more reliable indicators of power-law structure, but also suggests the lower threshold of site identification masks some legitimate power-laws. If we imagine a scenario in which every isolated artifact or feature was recorded as a site, we would vastly increase the number of small sites and more accurately capture power-law structure by incorporating all those locations used only once or a few times for a total occupation intensity corresponding to only a few person hours. The practicalities of archaeological survey make this unrealistic, but we should not take the failure to find power-laws in some contexts as evidence of their absence without first considering methodological constraints. These observations are most notable in that they suggest missing many smaller sites may actually be a bigger issue than definitional criteria that determines the boundaries of larger sites, which is where most prior concern and discussion has been directed.

Site level analysis alternatives

Throughout the analysis portion of the principal text, we simplify our discussion by focusing on only the loci approach to site boundary definition. In most cases, results would be strengthened by considering the sample with all loci amalgamated. Most sites are single loci, so this is an alternative approach that only affects a few of the largest sites: Ojo de Iitoi, Papago Tanks, Chivos, and Tule and two relatively modest sites Las Dunas and Suvuk. The figures provided here offer this alternative data analysis approach. As all conclusions and interpretations remain fundamentally unchanged and in several cases are strengthened, there is little need for additional discussion.

S2 Table presents correlation coefficients and other statistical parameters for a log log analysis of the data. This follows the methods of settlement scaling literature [3-6]. As with Fig. 6 in the principal text, it can be seen that a log-log plot of area vs ground stone counts reflects a much stronger relationship in the Las Playas data than in the Pinacate data which includes sites with more complex occupation histories. Statistically significant results are obtained for the Pinacate-only data for a site level analysis. More informative are the slope coefficients which are far below those predicted by Settlement Scaling theory for the Pinacate data, but higher than expected for the larger amalgamated sample. The latter is in keeping with prior explorations of hunter gatherer data as discussed in the principal text, while the former we argue to reflect the dominance of site reuse in the structuring of site assemblages.

In regard specifically to differences between site vs loci level analysis, there is little difference. As with the loci data presented in the principal text, the site level data presents a strong correlation between ground stone and structures and a more or less equally poor correlation between area and both material proxies (Figs 7-9). The one point of notable departure is that treating Papago Tanks as a singular site facilitates a more straight-forward interpretation of its unique status visible in the lack of material remains relative to its size. This is somewhat more pronounced in relation to structures. As discussed in the principal text, this is almost certainly related to Papago Tank’s status as a common pool resource with unique etiquettes regarding the proximity of camps in a multi-ethnic setting. Papago Tanks likely reflects a truly integrated site with some partitioning of space by function whereas other multi-loci sites reflect redundant use of space corresponding to frequent reuse that did not typically simultaneously encompass the entire site area. For this reason, we feel it is more appropriate to treat the data with the loci level approach.

SI Supporting Information References

1. Gravel-Miguel, C., J. K. Murray, B. J. Schoville, C. D. Wren, and Marean C. W. Exploring variability in lithic armature discard in the archaeological record. J Hum Evol. 2021;155:102981.
2. Haas RJ, Klink CJ, Maggard GJ, Aldenderfer MS. Settlement-Size Scaling among Prehistoric Hunter-Gatherer Settlement Systems in the New World. Plos One. 2015;10:e0140127. doi:10.1371/journal.pone.
3. Ortman S, Scheiber LL, Cooper Z. Scaling Analysis of Prehistoric Wyoming Campsites: Implications for Hunter-Gatherer Social Dynamics. In: Clark A, Gingerich JAM, editors. Intrasite Spatial Analysis of Mobile and Semisedentary Peoples. Salt Lake City: University of Utah Press; 2022. p. 109-22.
4. Ortman SG, Lobo J, Smith ME. Cities: Complexity, theory and history. Plos One. 2020;15(12):e0243621.
5. Lobo J, Whitelaw T, Bettencourt LM, Wiessner P, Smith ME, Ortman S. Scaling of hunter-gatherer camp size and human sociality. Current Anthropology. 2022;63(1):68-94.
6. Hamilton MJ, Buchanan B, Walker RS. Scaling the size, structure, and dynamics of residentially mobile hunter-gatherer camps. American Antiquity. 2018;83(4):701-20.


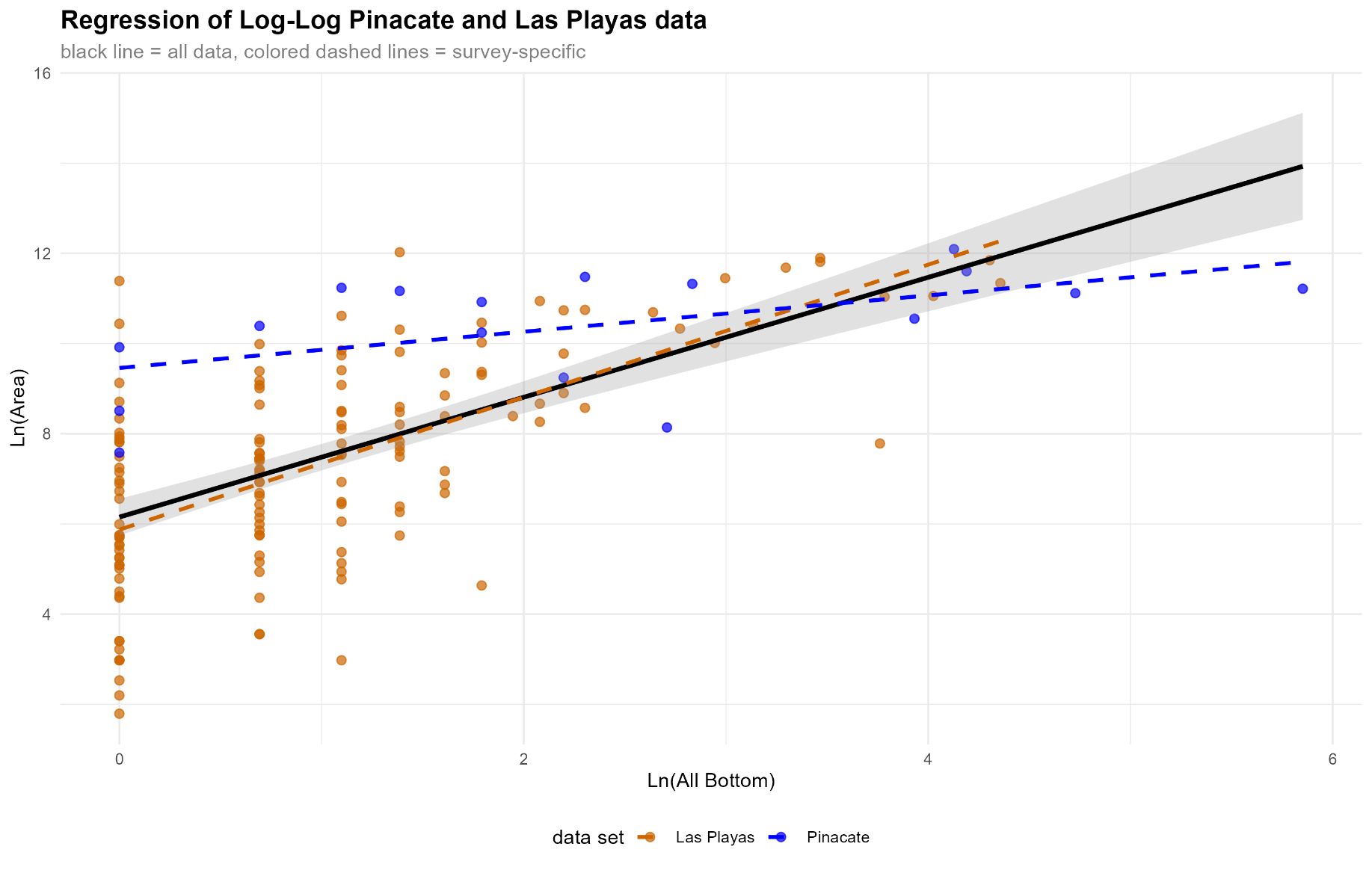


**S1 Fig. Regression Analysis of Survey Data.** The plot visually demonstrates the much improved fit between area and count of bottom stones of the Las Playas data set compared to the Pinacate data set. In contrast to Fig 6, this data is based on sites as opposed to loci. This data is log-transformed.


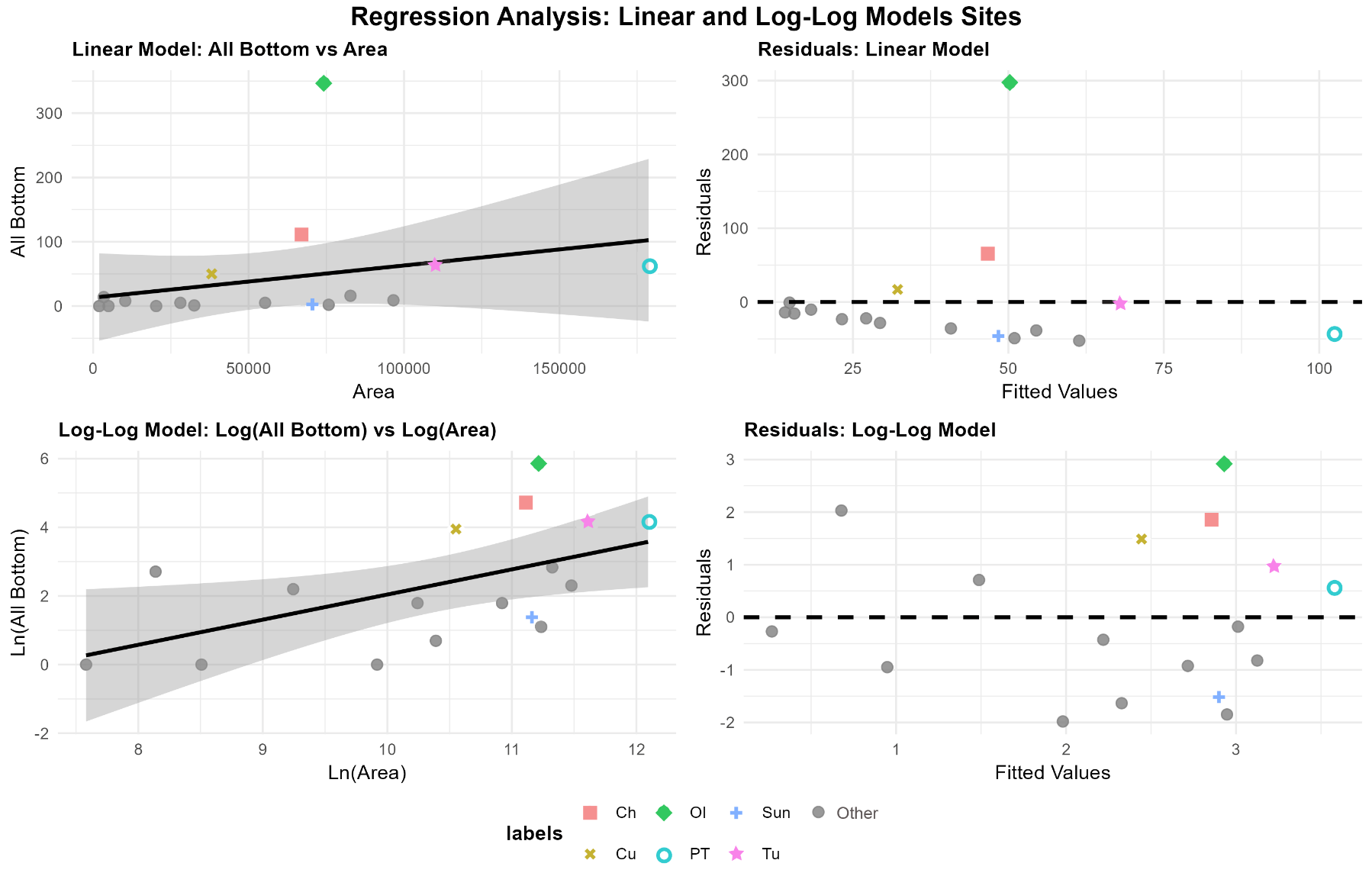


**S2 Fig. Linear and Log-Log Regressions with Residuals vs. Fitted Values.** This plot explores the relationship between site area and count of bottom stones. In contrast to Fig 7, this data is based on sites as opposed to loci. Abbreviations: Ch-Chivos, Cu-Cuervos, OI-Ojo de Iitoi, Papago Tanks-PT, Sun-Sunset, NA-all other sites.


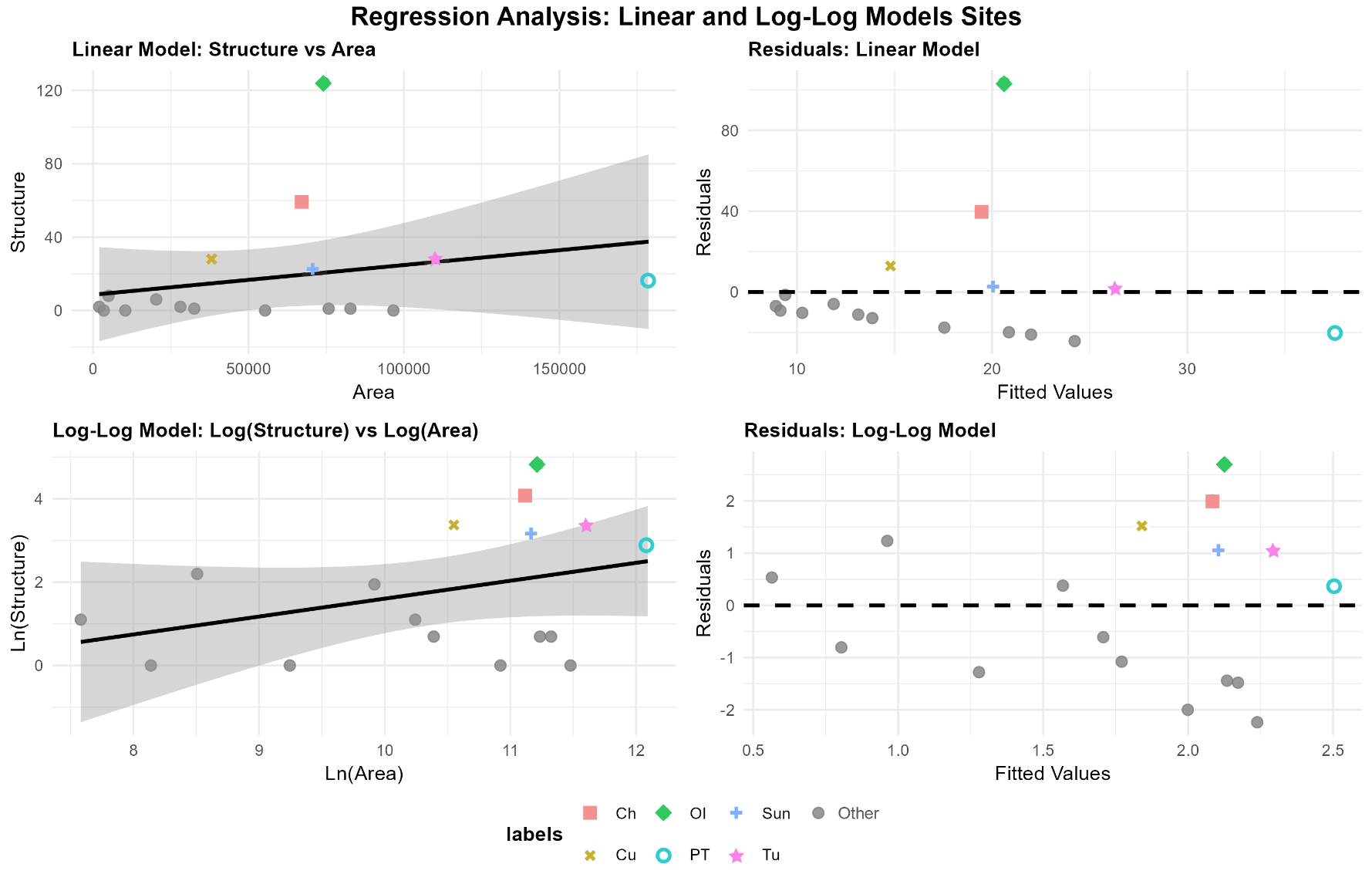


**S3 Fig. Linear and Log-Log Regressions with Residuals vs. Fitted Values.** This plot explores the relationship between site area and count of structures. In contrast to Fig 8, this data is based on sites as opposed to loci. Abbreviations: Ch-Chivos, Cu-Cuervos, OI-Ojo de Iitoi, Papago Tanks-PT, Sun-Sunset, NA-all other sites.


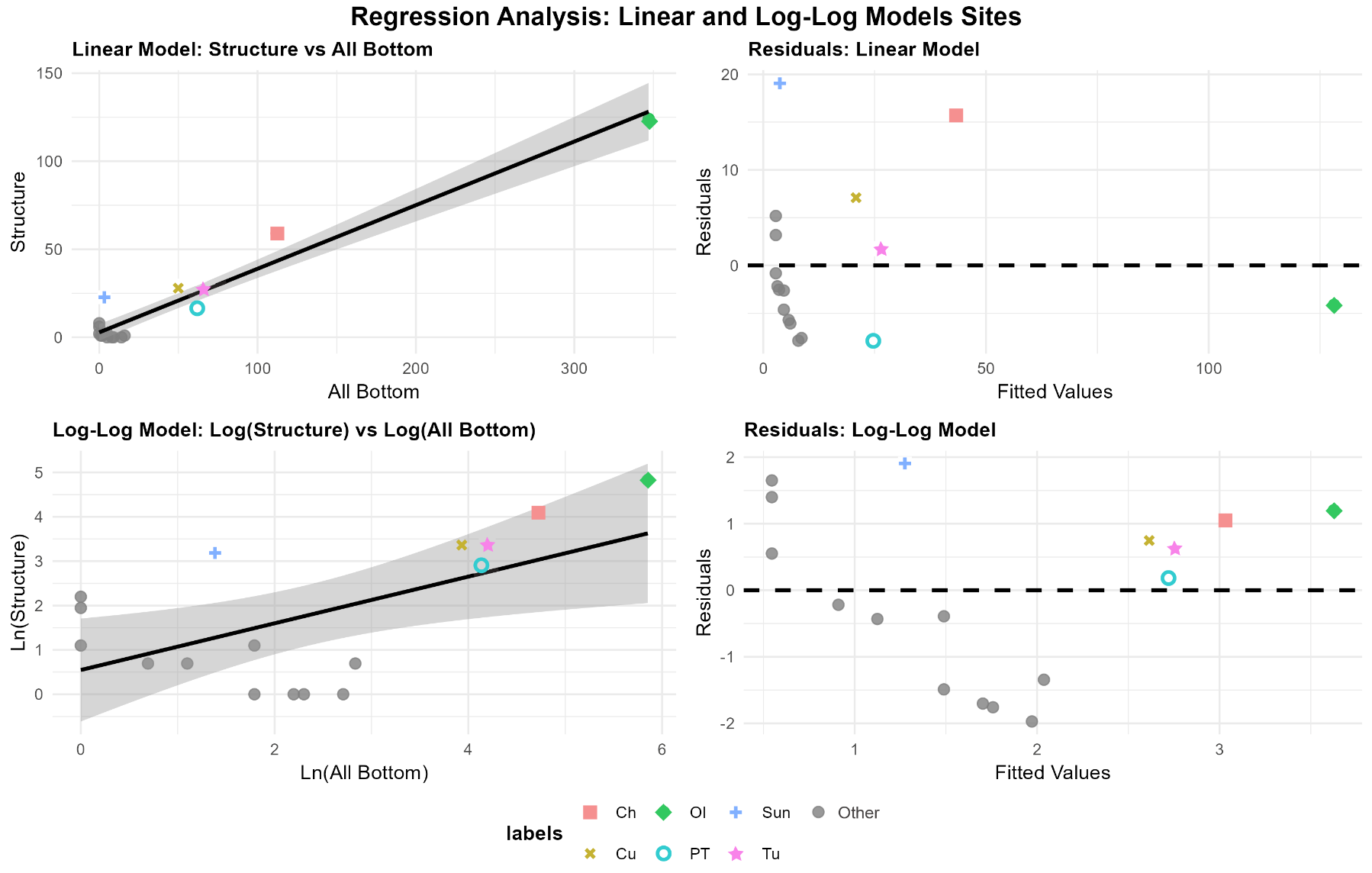


**S4 Fig. Linear and Log-Log Regressions with Residuals vs. Fitted Values.** This plot explores the relationship between count of structures and count of bottom stones. In contrast to Fig 9, this data is based on sites as opposed to loci. Abbreviations: Ch-Chivos, Cu-Cuervos, OI-Ojo de Iitoi, Papago Tanks-PT, Sun-Sunset, NA-all other sites.

**S1 Table. Correlation Coefficients for Alternative Approaches to Quantifying Ground Stone.**

| Loci r^2^ |  |  |  |  |  |
| --- | --- | --- | --- | --- | --- |
|  | area m2 | total structure | all ground stone | all bottoms | sum bottoms |
| area m^2^ |  |  |  |  |  |
| total structure | 0.02 |  |  |  |  |
| all ground stone | 0.01 | 0.77 |  |  |  |
| all bottoms | 0.01 | 0.77 | 1 |  |  |
| sum bottoms | 0.01 | 0.69 | 0.96 | 0.96 |  |
| sum wear mm | 0.01 | 0.76 | 0.98 | 0.96 | 0.96 |
|  |  |  |  |  |  |
| Site r^2^ |  |  |  |  |  |
|  | area m2 | total structure | all ground stone | all bottoms | sum bottoms |
| area m^2^ |  |  |  |  |  |
| total structure | 0.06 |  |  |  |  |
| all ground stone | 0.08 | 0.94 |  |  |  |
| all bottoms | 0.07 | 0.94 | 1 |  |  |
| sum bottoms | 0.14 | 0.88 | 0.98 | 0.98 |  |
| sum wear mm | 0.1 | 0.94 | 0.98 | 0.98 | 0.96 |

**S2 Table. Results of Regression and Correlation Analysis of Log-Log Data, Iterated by Site and Loci and by Data Set.**

| Data set | Loci/Site | x | y | intercept | slope | r2 | r | df | p-value | 95 low | 95 high | T |
| --- | --- | --- | --- | --- | --- | --- | --- | --- | --- | --- | --- | --- |
| Pinacate | Loci | ground stone bottom | area | 9.304 | 0.276 | 0.12 | 0.346 | 25 | 0.077 | -0.039 | 0.642 | 1.845 |
| Pinacate, Las Playas | Loci | ground stone bottom | area | 6.232 | 1.273 | 0.4 | 0.633 | 169 | **<0.001** | 0.534 | 0.715 | 10.64 |
| Pincate, Las Playas, Coastal | Loci | ground stone all | area | 6.058 | 1.264 | 0.45 | 0.671 | 175 | **<0.001** | 0.581 | 0.744 | 11.96 |
| Pinacate | Loci | structure | area | 9.675 | 0.157 | 0.03 | 0.184 | 25 | 0.359 | -0.211 | 0.527 | 0.934 |
| Pinacate | Loci | ground stone bottom | structure | -1.476 | 0.485 | 0.21 | 0.453 | 25 | **0.018** | 0.088 | 0.711 | 2.539 |
| Pinacate | Site | ground stone bottom | area | 9.458 | 0.402 | 0.34 | 0.543 | 15 | **0.024** | 0.841 | 0.812 | 2.503 |
| Pinacate, Las Playas | Site | ground stone bottom | area | 6.151 | 1.329 | 0.42 | 0.647 | 159 | **<0.001** | 0.547 | 0.729 | 10.698 |
| Pinage, Las Playas, Coastal | Site | ground stone all | area | 5.957 | 1.317 | 0.47 | 0.689 | 165 | **<0.001** | 0.599 | 0.761 | 12.196 |
| Pinacate | Site | ground stone bottom | structure | 1.177 | 0.651 | 0.34 | 0.585 | 15 | **0.014** | 0.146 | 0.832 | 2.796 |
| Pinacate | Site | structure | area | 9.878 | 0.292 | 0.13 | 0.354 | 15 | 0.163 | -0.153 | 0.713 | 1.466 |

**S3 Table. Raw Data of Ground Stone Counts, Structure Counts, and Area.**

<https://doi.org/10.5281/zenodo.17417738>
