# Supplementary material for: Exploring proxies for occupation intensity in hunter-gatherer settlement systems: A combination of ethnohistoric and archaeological data
Source: PLoS One. 2025 Nov 7;20(11):e0333870. doi: 10.1371/journal.pone.0333870 (PMC12594338; doi:10.1371/journal.pone.0333870)
Supplement: Attachment — Submitted filename: Review.docx [file pone.0333870.s003.docx]

In this study, Clark et al investigate how well different archaeological proxies reflect occupation intensity in hunter-gatherer settlement systems, using data from the traditional homeland of the Hia-Ced O’odham in the Sierra Pinacate and adjacent regions of Sonora, Mexico. Drawing on both archaeological survey data and ethnohistoric records, the authors assess how features such as site size, ground stone artifact counts, and windbreak structures relate to occupation intensity, conceptualized as a combination of group size, duration of stay, and frequency of reuse.

They find:

1) Ground stone counts and structure counts are robust proxies for occupation intensity, whereas site area shows only a weak and inconsistent relationship.
2) Occupation intensity across the landscape follows a power-law distribution, consistent with theories of preferential site reuse.
3) Divergences between site size and material proxies are not seen as confounding, but rather as analytical opportunities to infer site-specific roles in broader settlement systems.
4) Ethnohistoric insights help interpret these divergences, revealing factors like ritual use, common-pool resource dynamics, and inter-group cooperation/conflict that influenced site use.

By combining detailed regional archaeology with theoretical modeling and ethnohistoric context, the authors suggest that deviations in proxy relationships can yield nuanced reconstructions of landscape use, especially in systems featuring mixed forager-collector strategies.

The data are interesting and some of the results are quite suggestive (see Figure 5, for example). Others are less compelling. The correlations in Figure 7 are hard to interpret as the majority of the data is located to the lower left quadrant, and most clustered near zero. This both anchors the data artificially and increases undue leverage as we move up the x and y axes. The data point in the upper right (Od13) clearly has undue leverage here. This means the data need to be logged (and then you will find similarly strong, but more robust results).

There are also minor issues with terminology: for example, in line 547 the authors state “The site of Sunset 547 occupies a notably **supra-linear** position in this relationship” and in line 563 “Papago Tanks as individual loci or a unified site, it retains a 564 notable **sub-linear** status”. The authors here are referring to positive or negative residuals. The terms supra- or sub-linear refer to linearity of the fits (i.e., the slopes of regressions), not to the deviation of individual data points from their expected values.

My more major concern with the manuscript in this draft is the clarity of the writing. The paper starts quite strongly with a solid introduction to the issue of hunter-gatherer mobility (but there are more papers to cite here) and highlights the lack of direct archaeological proxies. This is great and unquestionably true.

However, once the authors get into the Data Collection Methods and Goals and beyond, the paper becomes increasingly hard to follow. Different kinds of data were collected from different sites (this seems justifiable) but it is difficult for the reader to follow this, and the variables are given entirely non-intuitive names “all bottoms”, “sum bottoms”, “all ground stone”. As such, it is very hard to keep these straight. And this goes further into the Proxy Quantification section. By the time we reach Survey Results, I am already confused.

It is also unclear why the Analysis and Discussion sections are lumped together. The authors start with a discussion of the workflow and present results, which seem to be interspersed with interpretations, caveats, and qualifiers. Again, this makes it hard to follow.

The section Assessing the representativeness of the settlement pattern I found particularly hard to follow. As someone familiar with these types of analysis, I found the presentation a little convoluted and confusing. In particular, I did not follow lines 474 to 485. There are various statistical methods of dealing with zero-inflated data, but, although widely practiced, adding “1” to all values when zeros are real data has no statistical meaning, especially when dealing with data on these logarithmic scales. By the time we reach the Conclusions, I was not overwhelmed by their support from the data, or exactly what I should have learned.

In sum, many of the individual sections need serious editing and trimming as they are not clear. Mu suggestion would be to simply focus of the strongest results in Figure 5 and tell us about their importance. Remove much of the other analysis and description to Supplementary Materials. At the moment, there seems to be a very simple and perhaps compelling story to tell here from this case study, but currently that message is buried under quite an opaque text.
