# Supplementary material for: Exploring proxies for occupation intensity in hunter-gatherer settlement systems: A combination of ethnohistoric and archaeological data
Source: PLoS One. 2025 Nov 7;20(11):e0333870. doi: 10.1371/journal.pone.0333870 (PMC12594338; doi:10.1371/journal.pone.0333870)
Supplement: Attachment — Submitted filename: Response_to_Reviewers.docx [file pone.0333870.s005.docx]

Response to Reviewers:

We note that the two reviewers to some extent request alternative and not entirely resolvable approaches to improvement. One suggests a variety of new analysis, or at least raise issues that require additional analyses. The other suggests a winnowing to a few key observations. We attempt to negotiate this with fidelity to the core concerns of each reviewer and note both had concerns for readability and clarity. To address this shared concern, we have moved a number of parenthetical and tangential discussions to the supplemental material as requested by Reviewer 2 and in other locations simply remove minor points.

Reviewer 1 provides a number of very insightful and useful suggestions. Most broadly they invite us to tie our research to other recent discussions of hunter and gatherer settlement patterns that focus on the relationship between demography (inclusive of occupation intensity) and site size. We have endeavored to do this throughout the paper, most overtly in the theoretical background section and the discussion. We also add a new analysis section that considers this issue as well as addressing the suggestion to explore log transformed data. However, it is also important to highlight we are dealing with a different sort of sample and attempting to address some distinct questions that depart from the goals of those addressed in the recommended citations that focus on Settlement Scaling Theory (SST). We do not intend to critique Settlement Scaling Theory, but this perspective has most often presented hunters and gatherers as a useful comparison to sedentary groups, whereas our concerns are fundamentally about developing methods to discern variation within hunter and gatherer settlement systems. There are key distinctions in the sort of distribution we are working with and those employed by SST applications. Most importantly, our sites have a much more time averaged character. We made this point obliquely in the initial paper and attempted to clarify more directly in this version. Our data would not be appropriate for inclusion in most analysis of SST because we would never present it as representing momentary maximums of occupation. While this may be seen as a detriment from the perspective of SST, it is by far the more common character of hunter and gatherer settlement pattern data and underpins our primary goal of advancing discussions of unpacking the concept of occupation intensity. It might be useful to note that one primary author studies the settlement patterns of the Paleolithic while the other works with much more recent groups. The goals of this paper are very much intended to direct attention at the sorts of mobility/landscape/group organizations issues that have purchase across this spectrum of comparative context.

We have attempted to demarcate in the first two sections of the paper the points where SST and occupation intensity discussions overlap and also where they depart. To the extent that much of our data looks suspect to Reviewer 1, we think it requires no more explanation than that our data is the result of many occupations and does not intend nor claim to capture momentary maximum population. For example, the reviewer rightly directs us to the relevance of the Ortman et al. 2022 chapter (of which one of us was an editor). Here, the authors go to significant lengths to demonstrate that site artifact densities present essentially no correlation with structure counts (teepee rings) to verify the latter largely capture the largest momentary population as opposed to an accumulated occupation history. A point further upheld with significant ethnographic contextual discussion. Our data is a virtual mirror image of this. The high correlation between artifacts and structures suggest they are both cumulative records, a point which we discuss at length. We would, of course, be thrilled if other scholars used our data to extend SST investigations into more time averaged deposits through novel approaches, but that is another question for another day.

In this revised version we likely steer the discussion in a different direction than that envisioned by Reviewer 1’s suggestion, but we hope our revisions will meet with approval and reflect our concerted effort to identify fruitful points of theoretical overlap. Reviewer 1 made a variety of suggestions that broadly can be taken as attempts “to fix” our data so that it reflects prior empirical observation and theoretical predictions of SST. We instead believe Reviewer 1 has identified another and perhaps more fruitful path than our original approach to identify site occupation histories dominated by site reuse. While this does not seem to be a central focus of Reviewer 1’s interests, it is of fundamental importance to hunter and gatherer archaeology.

Specific points of revision

Reviewer 1 suggests we consider log-normal distributions in addition to Power Law, geometric and Poisson in assessing the distribution of ground stone. Log-normal distributions are continuous and thus cannot be appropriately applied to discrete (count data). That being said, Reviewer 1 is absolutely correct that the artifact and feature distributions have the general form of a log-normal distribution. Coercing the data in R to test this does indicate that the Pinacate (but not the Las Playas or combined data) plausibly follows this pattern. However, again it is technically not good practice to include this distribution among the options. We also emphasize that our intent with the distribution fit model is closely modeled on Haas et al. to demonstrate that we are dealing with the same sort of data structure to make our arguments comparable. Exploring fits to other distributions is interesting, but somewhat of a side quest. We have adjusted the language and the order in the discussion where this analysis is situated to clarify its role in the larger presentation.

Reviewer 1 requests a statistical analysis based on log transformed variables. We provide this as requested along with the relevant statistical estimations. As Reviewer 1 noted from their own experiments with our data--something we much appreciate--we produce slopes (exponents) that depart significantly from the predictions of SST or prior investigations of momentary maximum populations of hunter gatherer sites. As we explain in text, we believe the magnitude of departure captures the relative degree to which site re-use contributes to overall occupation intensity. As we discuss this substantively in text we will not delve into more details here.

Reviewer 1 questioned whether there might be a biasing factor in the definition of site boundaries and whether any further analysis could be provided to eliminate such a bias. We understand this concern, but we’re not sure what more could be practically done to mitigate against this form of error. The reviewer is likely aware that this concern is raised in almost all of the recommended references as well as in the Haas et al article that we closely follow. We note that our alternative approaches that explore both loci and site definitional approaches was intended to explore this issue and thus we have already provided a rather exhaustive alternative set of models. Specifically, the Loci approach essentially asks what if we consider some of these larger sites as actually a series of smaller sites broken into their densest components. This is the model we generally prefer (as we feel it is a more accurate representation of the data) despite the fact that we often get “better” statistical results with the non-Loci approach. We hope this demonstrates are commitment to presenting the data as honestly as possible with all of its flaws apparent. More broadly, there does not seem to be any basis for Reviewer 1’s specific concern that larger sites have a lower threshold for their outer boundary. We endeavored to be consistent in the field in mapping site boundaries. We acknowledge there is always some level of subjectivity, however, there seems to be no basis for suggesting that our large sites had more poorly defined boundaries other than it might push our distributions in the direction of meeting the expectations of SST. We can agree there is a need to explain this departure, which as already noted, we believe reflects the Pinacate sites’s complex occupational histories in which site boundaries do not capture the momentary maximum population.

Reviewer 2 provided a number of useful comments. Many were necessarily addressed or obviated in the revisions undertaken in response to Reviewer 1 and thus we will not itemize these again here. The most overt concerns of Reviewer 2 were more general comments on readability and organization. Specifically, they encourage us to limit interpretations to only a few key points and to reduce the amount of contextual background. Hopefully, it is evident that we prioritized these concerns within the bounds of addressing other suggestions.

The question of zero values raised by Reviewer 2 is noted. We are cognizant of the admonishment that simply adding a value of one to data sets, though common, is not always justifiable. Our imperfect response is that much of the significance of our approach is predicated on replicating prior applications and their methods. Most of the more mathematically elaborate methods of dealing with zeros entail running alternative statistical tests which would have no comparable baselines in the well cited literature. We note that we already explored a few other approaches in our initial submission and attempted to explain why we opted to retain the simple add-one across the entire distribution method. We moved this discussion and the related parenthetical observations to the supplementary material because we agree that it clouded the discussion. We now also include a brief discussion of our (and other’s) logic that these methods are intended to identify proxies of human time. In this sense, zero values are an issue of “sampling zeros” rather than “true zeros” and the adding of a one to the entire distribution is a relatively justifiable approach.

Point-by-point response (our response in italics):

Reviewer #1: The authors tackle an interesting issue in the analysis of hunter-gatherer settlement data. In many agrarian systems site area has a strong relationship with population proxies. But in H/G systems, site area is less well correlated with other measures of occupational intensity. The authors find the same here and discuss possible explanations for it.

There is a huge literature that examines the relationship between momentary population and area across permanent settlements, and the patterns are very clear there, as exemplified by Ortman et al. 2020. Discussing the regularity of these patterns would help to motivate the current work with hunter-gatherer data.

*References added.*

Regarding the distributional fits, I would think testing for a log-normal distribution would also be appropriate given that the upper tails of power and log-normal distributions are often very similar and difficult to distinguish from each other. I would add that to the list of MLE tests.

*Discussed above, count data is inappropriate for a log-normal distribution fit test. Additionally the point of this analysis is to demonstrate similar data structure to Haas et al. However, Reviewer 1 is absolutely right about the general structure of the data.*

I was also surprised not to see comparisons between area and features/artifacts using scaling analysis, with linear fits to log transformed variables. This is the more standard approach in the recent literature, and it allows one to account for nonlinear correlations between variables.

*We have now used log transformed variables as well.*

I couldn’t tell from Table 1 and the associated text which of the datasets is being used there. I think it must be Pinacate because these are the only data in the appendix that have area, structures, and groundstone tabulated at both the site and locus level. This should be clarified.

*We have clarified the different datasets and when they are used.*

The scatterplots are also pretty drafty looking and need to be improved for legibility.

*We have changed our scatterplots and endeavored to make them more legible.*

Once I looked at the data in the associated zip file I understood the analyses much better, but I have to admit that in reading the paper I often had difficulty telling which data were being discussed in which case. In fact, it took me awhile to even realize that there were different subsets of data that were being analyzed in different cases. It would be good for the authors to work on clarifying the language and presentation to make that more clear.

*We now begin our “analysis and discussion” section with an explanation of the datasets being used. We have also moved some of the analysis to the supplemental material. Our initial purpose in including alternatives was to show the results were robust even if different assumptions were made about site boundaries.*

The relationships between total structures, and total ground stone, vs. site area are really different depending on which dataset is used. For example, in their all regional dataset, the scaling relationship between total ground stone and total area (slope of the fit line on a log-log plot) has a slope of about 1; but in their Pinacate loci dataset, even when I add together all structures and all ground stone and compare that to site area, I get a slope of about .25 in the scaling relationship. So, in the regional data, site area increases roughly proportionately with the amount of material on the site, but in the Pinacate data the amount of material increases much faster than the area.

*We did point this out in our initial analysis and note it as reflective of the Pinacate data being more logistical in character. We now make this a central point of discussion and specifically note its contrast to SST predictions.*

It would be worthwhile for the authors to put a bit more effort into comparing their results with other studies of hunter-gatherer settlement data. In addition to the study by Haas et al. that the authors focus on, Lobo et al. and Hamilton et al. look at the relationship between population and area across ethnographic camps, and Ortman et al. examine the relationship between stone circle count and area in Wyoming stone circle sites. There is variation in these relationships across cases, but the slope is never as low as .25.

*Substantially more discussion on the relation to SST focused literature has been added. However, it is also important to recognize that the focus of SST is distinct from the more basic issues of occupation intensity. That is, our goals are not to advance SST but the broader and more established literature on occupation intensity.*

One simple possible explanation is site boundary definitions, such that density thresholds that define site edges decrease as the site size increases. It would be good to rule this out somehow.

*We added a discussion on how site boundaries were defined. We also already included a completely alternative set of analysis with a different approach to definition in the original paper. We feel there was no basis for Reviewer 1 to make this assertion other than it might make the data behave more similar to the predictions of SST, for which we have offered an alternative explanation.*

Alternatively, one might not expect total structures or artifacts to be proportional to the average momentary populations of sites, and the authors acknowledge this by structuring their discussion around occupational intensity instead of population per se. Something that occurs to me is that in this system more areally-extensive sites might be used by more people but much less frequently, during periodic aggregations only when conditions permit; whereas smaller sites might be used much more frequently or for longer durations by smaller groups. The density of ground stone definitely decreases with site area in the all regional data, so overall larger sites are used less intensively. This would seem to imply that smaller sites were used MUCH more often than larger sites. I suppose it is also possible that the accumulation rate of features and artifacts per person day of use decreases as the group size increases, as might occur if large groups gathered only infrequently and for short times relative to smaller groups. Lobo et al. discuss a model along these lines that might well fit this resource limited environment, where there is an intrinsic energy deficit involved in camping together in large groups, but people still want to gather when they can for social production, courtship, trade, information sharing, etc. Features and groundstone might then accumulate at slower rates per person day in larger sites because people are living with an energy deficit when camping together in large groups. These are just ideas for the authors to consider.

*Thank you, this is all very interesting and we’ve used these insights throughout our revised draft.*

Overall, I think this is an interesting dataset and I appreciate the authors’ careful conceptualization of the factors involved in the variation observed. In addition to considering the reactions above, I think the main thing the paper could use is a bit more organizational work on the discussion of the actual analyses. The background material is interesting, but the analysis write up is harder to follow. I could eventually figure it out, but I am putting more work into the paper as a reviewer than the average reader will.

*We’ve focused a lot of effort on clarifying our text and explaining our analyses more clearly.*

Reviewer #2: In this study, Clark et al investigate how well different archaeological proxies reflect occupation intensity in hunter-gatherer settlement systems, using data from the traditional homeland of the Hia-Ced O’odham in the Sierra Pinacate and adjacent regions of Sonora, Mexico. Drawing on both archaeological survey data and ethnohistoric records, the authors assess how features such as site size, ground stone artifact counts, and windbreak structures relate to occupation intensity, conceptualized as a combination of group size, duration of stay, and frequency of reuse.

They find:
1) Ground stone counts and structure counts are robust proxies for occupation intensity, whereas site area shows only a weak and inconsistent relationship.
2) Occupation intensity across the landscape follows a power-law distribution, consistent with theories of preferential site reuse.
3) Divergences between site size and material proxies are not seen as confounding, but rather as analytical opportunities to infer site-specific roles in broader settlement systems.
4) Ethnohistoric insights help interpret these divergences, revealing factors like ritual use, common-pool resource dynamics, and inter-group cooperation/conflict that influenced site use.

By combining detailed regional archaeology with theoretical modeling and ethnohistoric context, the authors suggest that deviations in proxy relationships can yield nuanced reconstructions of landscape use, especially in systems featuring mixed forager-collector strategies.

The data are interesting and some of the results are quite suggestive (see Figure 5, for example). Others are less compelling. The correlations in Figure 7 are hard to interpret as the majority of the data is located to the lower left quadrant, and most clustered near zero. This both anchors the data artificially and increases undue leverage as we move up the x and y axes. The data point in the upper right (Od13) clearly has undue leverage here. This means the data need to be logged (and then you will find similarly strong, but more robust results).

*We now use logged variables.*

There are also minor issues with terminology: for example, in line 547 the authors state “The site of Sunset 547 occupies a notably supra-linear position in this relationship” and in line 563 “Papago Tanks as individual loci or a unified site, it retains a 564 notable sub-linear status”. The authors here are referring to positive or negative residuals. The terms supra- or sub-linear refer to linearity of the fits (i.e., the slopes of regressions), not to the deviation of individual data points from their expected values.

*We’ve changed this.*

My more major concern with the manuscript in this draft is the clarity of the writing. The paper starts quite strongly with a solid introduction to the issue of hunter-gatherer mobility (but there are more papers to cite here) and highlights the lack of direct archaeological proxies. This is great and unquestionably true.
However, once the authors get into the Data Collection Methods and Goals and beyond, the paper becomes increasingly hard to follow. Different kinds of data were collected from different sites (this seems justifiable) but it is difficult for the reader to follow this, and the variables are given entirely non-intuitive names “all bottoms”, “sum bottoms”, “all ground stone”. As such, it is very hard to keep these straight. And this goes further into the Proxy Quantification section. By the time we reach Survey Results, I am already confused.

*We’ve invested significant effort to clarify our language. We’ve also moved some analyses to the supplemental material (like our analyses that determined which counts of ground stone are most reliable). We now only use only the bottom stones (i.e., metates, mortars, netherstones) in the analyses presenting in the main text which we hope eliminates the confusion.*

It is also unclear why the Analysis and Discussion sections are lumped together. The authors start with a discussion of the workflow and present results, which seem to be interspersed with interpretations, caveats, and qualifiers. Again, this makes it hard to follow.

*We have kept the analysis and discussion together but we’ve significantly streamlined and simplified the text so that its easier to follow.*

The section Assessing the representativeness of the settlement pattern I found particularly hard to follow. As someone familiar with these types of analysis, I found the presentation a little convoluted and confusing. In particular, I did not follow lines 474 to 485. There are various statistical methods of dealing with zero-inflated data, but, although widely practiced, adding “1” to all values when zeros are real data has no statistical meaning, especially when dealing with data on these logarithmic scales. By the time we reach the Conclusions, I was not overwhelmed by their support from the data, or exactly what I should have learned.

*Addressed above.*

In sum, many of the individual sections need serious editing and trimming as they are not clear. My suggestion would be to simply focus of the strongest results in Figure 5 and tell us about their importance. Remove much of the other analysis and description to Supplementary Materials. At the moment, there seems to be a very simple and perhaps compelling story to tell here from this case study, but currently that message is buried under quite an opaque text.

*By moving much of the supporting analyses to the supplemental material, we believe we have simplified the story and clarified the text.*
